# Case Report: Surgical Treatment of Severe Facial Wounds and Proptosis in a Dog Due to a Traffic Accident

**DOI:** 10.3389/fvets.2020.548279

**Published:** 2020-12-18

**Authors:** Jury Kim, Daesik Kim, Janghwan Kim, Daeyun Seo, Hyejin Hwang, Yuna Kim, Taekyu Chung, Seongsoo Lim, Hansol Lee, Min Su Kim

**Affiliations:** ^1^Bundang Bright-Eye Animal Hospital, Seongnam, South Korea; ^2^Veterinary Emergency Medicine, Department of Veterinary Clinical Science, College of Veterinary Medicine and Research Institute for Veterinary Science, Seoul National University, Seoul, South Korea

**Keywords:** canine, trauma, face injury, proptosis, skin flap

## Abstract

Although facial wounds caused by traffic accidents in dogs are common, the surgical management of severe facial injuries involving the soft tissue, bone, dentition, nose and orbit are challenging. A 2 year-old Korean Jindo dog was diagnosed with severe skin defects of the face and proptosis caused by a vehicular accident. Along the left lateral maxilla, severe injury involving the overlying skin and platysma muscle occurred, to the extent that the middle part of the sphincter colli profundus pars intermedia muscle was exposed. Repair surgeries of the skin defects and globe displacement were performed using a local subdermal plexus rotation flap and a partial transposition of the dorsal rectus muscle combined with small intestinal submucosa (SIS) instead of enucleation as the first attempt. SIS was used to sustain the torn medial region. In this case, the surgery resulted in good cosmetic and functional outcome in the dog, despite the atypical complexities upon presentation.

## Introduction

Facial trauma is a frequent and significant cause of poor functional prognosis (1). The management of facial wounds is generally complicated because they affect important structures, such as the mouth, nose, ear, and eye (2). Because soft tissue is limited compared to other parts of the body, simple closure techniques can be more challenging (3). Facial skin wounds can be repaired using locally accessible tissues through various techniques, such as an axial pattern flap or a subdermal plexus flap. If large defects have occurred involving the lips, or mucous membranes, grafts are often used for repair (2). The subdermal plexus flap has been used to repair acute or chronic wounds. For cases involving the maxilla, neck, and forehead, local subdermal plexus flaps, such as rotation, transposition, and advancement flaps, can be applied. Viability of the local subdermal plexus flap depends on the vascular supply at the flap base (4). Because of early vascularization of the subdermal vascular network, the local subdermal plexus flap easily survives and routinely has been used in various facial wounds (5).

In veterinary medicine, ocular injuries caused by traumatic accidents include corneal laceration, iris prolapse, and proptosis (6, 7). Ocular misalignment can occur due to orbital fracture, nerve damage, or muscular damage (8–10). Although anatomical restoration has been performed in humans, strabismus with ocular misalignment has been difficult to correct in dogs because of anatomical and technical limitations (11). If the eye and the extraocular muscles are intact, globe replacement with tarsorrhaphy should be performed. If the eye and most extraocular muscles are ruptured with infected tissue, enucleation is generally recommended (7). Small intestinal submucosa (SIS) in veterinary ophthalmology has been used to manage severe corneal damage and corneoscleral defects as newer grafting methods (12). However, to the best of the authors' knowledge, there are no reports using SIS as a support material for the extraocular muscle.

In the case presented, we considered whether the proptosis could be induced by damage to the medial rectus muscle (MRM). Ocular misalignment, such as strabismus or proptosis after trauma, may develop from orbital fracture, muscular damage, and/or nerve injury (9, 10). In dogs, acquired proptosis with damage of MRM may develop from proptosis secondary to blunt head trauma (11). It is a common sequela resulting from avulsion of the MRM, which is the shortest of the four rectus muscles in dogs and can reduce the vitality of the globe and cause loss of vision (13). Proptosis is an acute emergency condition, and immediate intervention is essential after the patient is stabilized (7, 11). Temporary tarsorrhaphy with globe reduction and enucleation are two treatment options. If the extraocular muscle is ruptured, dorsolateral strabismus of the eye can result (14). Surgical corrections are warranted. In humans strabismus can result in vision impairment, headaches, and fatigue. If successful, surgical intervention can result in improved quality of life. However, surgical correction of the strabismus caused by ruptured muscles has been reported to be very challenging (15). Attempts to reattach the severed ends of the MRM were not usually successful, as the deeper aspects of the MRM could not be located (13).

The purpose of this report is to describe a challenging case regarding the surgical management of both large facial skin defects and globe displacement using a local subdermal plexus rotation flap and a partial transposition of the dorsal rectus muscle combined with the SIS in a dog.

## Case Presentation

A 2 year-old female Korean native Jindo dog was referred to a local emergency hospital for severe facial trauma with large skin defects and OS proptosis. The dog had been involved in a traffic accident 2 days prior, which had caused severe facial wounds and proptosis of the left eye. The eye had been proptosed for approximately less than an hour. Prior to presentation the prolapsed eye was manually reduced by placing pressure on the globe with the handle of a scalpel. Basic wound care consisted of flushing debris away with sterile saline and applying daily bandages.

**Figure 1:**
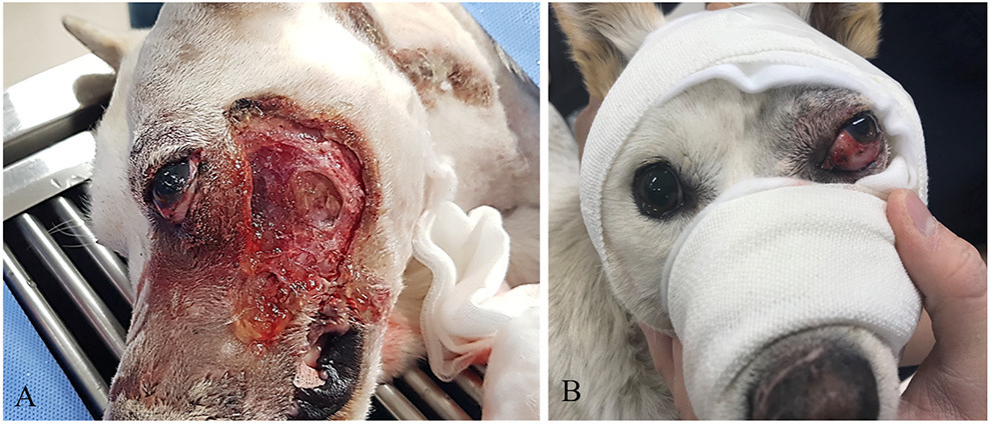
**(A)** Severe skin defect and necrotic debris. **(B)** Dorsolateral strabismus before surgical correction.

On physical examination, the dog presented with tachycardia and tachypnea. Other vital signs were unremarkable. Skull radiographs were normal, and no abnormalities were noted involving the dentition. However, along the left lateral maxilla, the external skin and middle part of the sphincter colli profundus pars intermedia muscle were exposed (Figure 1A). Ocular examination of the left eye revealed moderate periocular swelling, conjunctival injection, and severe dorsolateral globe displacement with mild exophthalmos (Figure 1B). No abnormalities were observed in the right eye, including intraocular examination by slit lamp biomicroscopy and indirect ophthalmoscopy. Neither cornea appeared damaged based on fluorescein dye staining OU. The trigeminal and facial nerves involving both sides of the face were presumed to be intact, based on normal palpebral reflexes and vibrissae responses. It was difficult to determine a clear menace response in the left eye because of the globe displacement. Vestibuloocular reflexes appeared positive for the right eye, but accurate assessment was difficult because of the periocular swelling. Direct and indirect pupillary light reflexes (PLR) and dazzle reflexes were positive in both eyes.

The owner consented to surgical management of the skin and ocular damage. The surgical plan included a local subdermal plexus rotation flap, repair of the medial rectus muscle (MRM) using the SIS patch, and partial transposition of the dorsal rectus muscle (DRM) above the SIS. Three days were required to prepare a healthy granulation bed within the wound and commercial SIS.

Open wound management with twice daily soaking with sterile saline soaked gauze was performed before surgery. Temporary tarsorrhaphy was performed on the globe for protection until a more definitive surgery could be performed. Topical artificial tears, amoxicillin-clavulanic acid (13.75 mg/kg PO twice daily), meloxicam (0.1 mg/kg PO once daily) and tramadol (3 mg/kg PO twice daily) were prescribed for 6 days before surgery.

Seven days after the initial trauma, healthy granulation tissue had developed along the left maxilla, and surgery was performed under general anesthesia. A complete blood count and biochemistry panel revealed no remarkable findings, except for hypoproteinaemia (4.9 g/dL; reference range, 5–7.4 g/dL) and increased aspartate aminotransferase (64 IU/L; reference range, 15–43 IU/L); the albumin was within normal range (2.8 g/dL; reference range, 2.4–4.4 g/dL). Metacam (Meloxicam inj.; Boehringer Ingelheim) at 0.2 mg/kg i.v., tramadol (Tramadol; Shinpoong) at 3 mg/kg i.v., and cefazolin (Cefazolin; Chongkundang) at 25 mg/kg i.v. were administered pre-operatively. General anesthesia was induced with 6 mg/kg propofol (Anepol; Hana Pharm Co., Ltd.) and maintained with sevoflurane (Sevofran; Hana Pharm Co., Ltd.) and oxygen on a circle system following endotracheal intubation.

**Figure 2:**
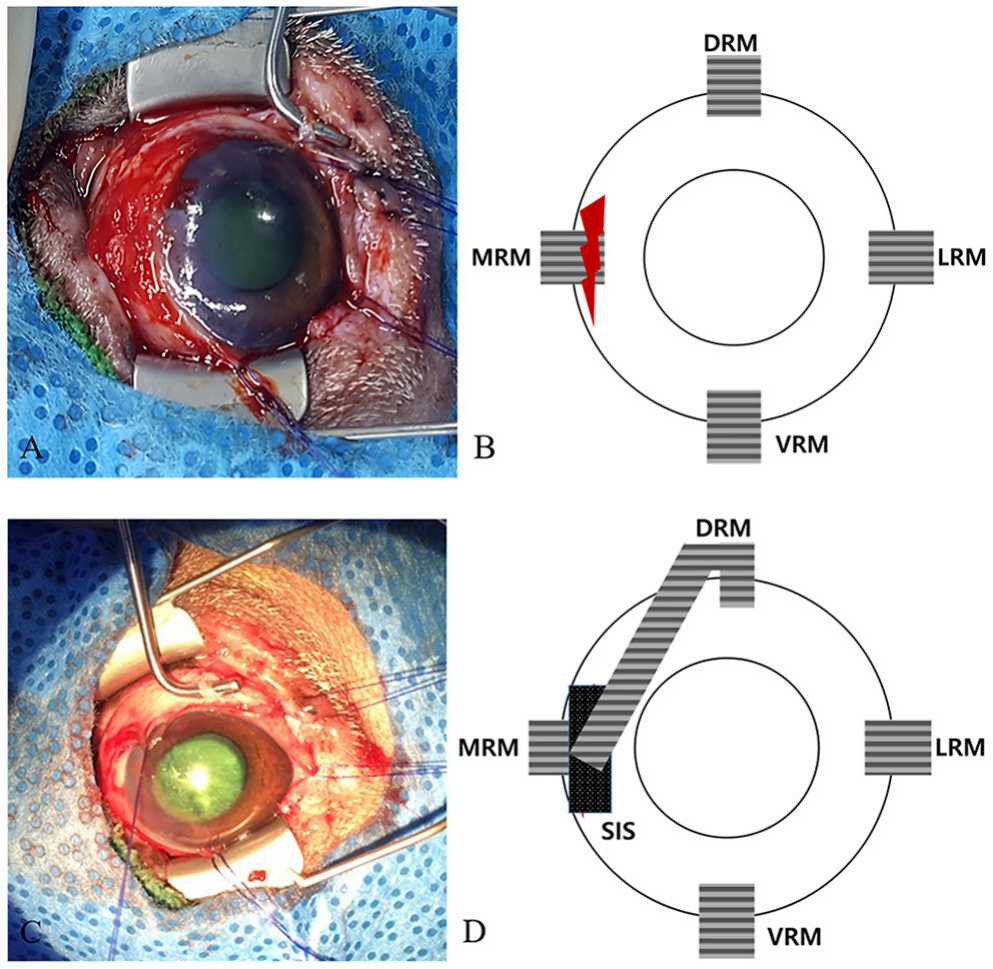
Surgical and schematic images of the strabismus repair. **(A)** Image of the initial ocular damage with the torn medial canthus muscle and sclera. **(B)** Schematic image of **(A)**. **(C)** Image of the sutures placed in the longitudinally split partial DRM and SIS patches above the torn area. **(D)** Schematic image of **(C)**. DRM, dorsal rectus muscle; MRM, medial rectus muscle; LRM, lateral rectus muscle; VRM, ventral rectus muscle; SIS, small intestine submucosa.

The patient was placed on its back in dorsal recumbency to confirm the symmetry of the eyes during surgery. The conjunctiva and protruded eyeball were lavaged with 0.9% sterile buffered saline solution (Normal Saline; JW Pharmaceutical). The MRM was not found to be damaged, but the sclera under the muscle was found torn, preventing a direct suture (Figures 2A,B). Therefore, partial transposition of the DRM was planned.

A commercial porcine SIS (small intestine submucosa; Vetrix BioSIS; Vetrix) patch was applied over the MRM using 8-0 Vicryl (coated vicryl; Ethicon) to reinforce the torn areas. Then the DRM was split longitudinally, transposed in the direction toward the SIS, and interrupted sutures were placed using 6-0 Vicryl (coated vicryl; Ethicon) (Figures 2C,D). A temporary tarsorrhaphy was performed using horizontal mattress sutures of 4-0 Vicryl (coated vicryl; Ethicon) for postoperative protection of the globe. The tarsorrhaphy procedure was performed, using a drawstring through the top and bottom bolsters, which were placed at the margins of the upper and lower eyelids.

**Figure 3:**
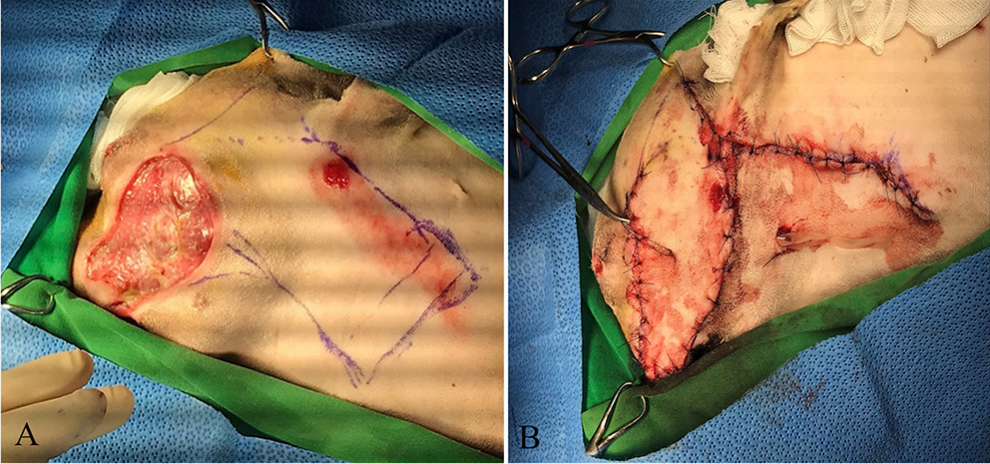
Reconstructive surgery of the facial skin defect. **(A)** Severe skin defect and necrotic debris before surgery. **(B)** The local subdermal plexus rotation flap at surgery.

**Figure 4:**
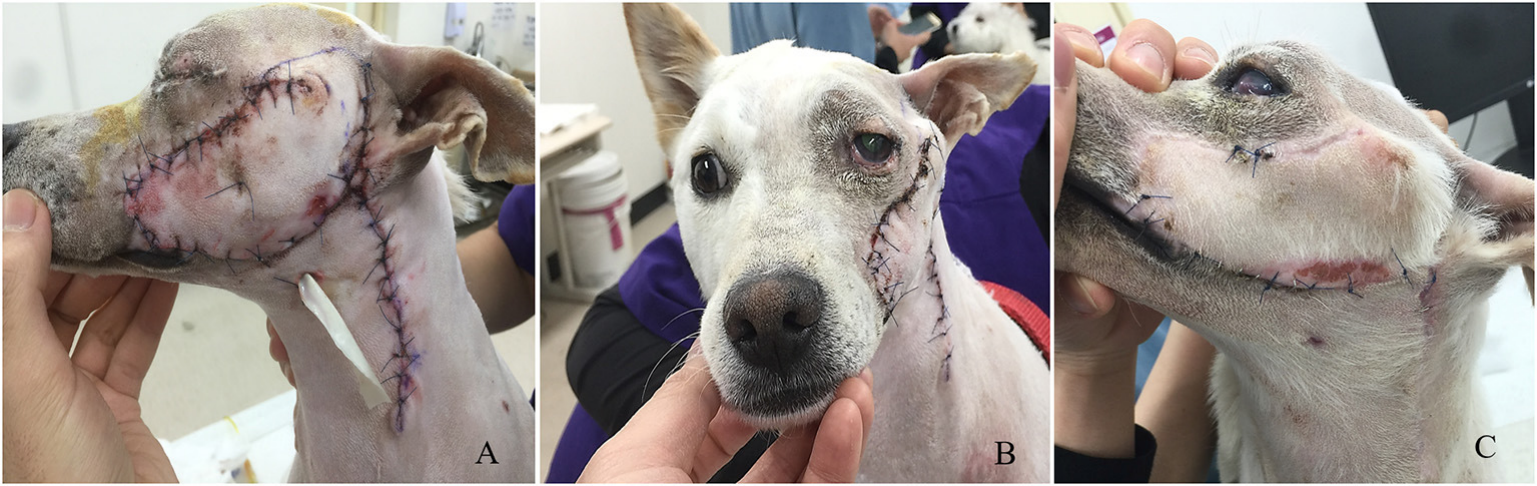
The changes of the flap after surgery. **(A)** Viability of the flap 1 day after surgery. **(B)** The conditions of the skin flap and globe 18 days after surgical correction. **(C)** The condition of the skin flap 21 days after surgery.

The patient was placed in right lateral recumbency. A local subdermal plexus rotation flap, 10 cm wide and 22 cm long, was applied (Figure 3A). The flap base extended from the palpable depression between the ventral ear canal to the lateral wound margins. The flap was sutured into the recipient site using 3-0 nylon in a simple interrupted pattern at the skin edges, and a Penrose drain was placed under the flap (Figure 3B). One day after the surgery, the subdermal plexus flap was viable, and the tarsorrhaphy appeared intact (Figure 4A). Postoperative medications included two antibiotic eye drops (tobramycin and moxifloxacin eye drops q8h for 10 days), artificial tear drops, systemic antibiotics (cefazolin 25 mg/kg p.o. q12h for 10 days), and two analgesics (tramadol 5 mg/kg p.o. q12h and meloxicam 0.1 mg/kg p.o. q24h for 10 days).

Serosanguinous discharge occurred postoperatively, gradually decreasing from soaking four 4 × 4 gauze to only one by day 4, at which point the Penrose drain was removed. The tarsorrhaphy was released 7 days after the surgery. Clinical assessment of the flap viability was performed at days 1, 5, and 18 after the surgery. At day one, the flap was mildly erythematous and edematous. At day 5, the erythema and edema resolved, and the skin appeared normal. At day 18, the hair was regrowing without any erythema or congestion.

**Figure 5:**
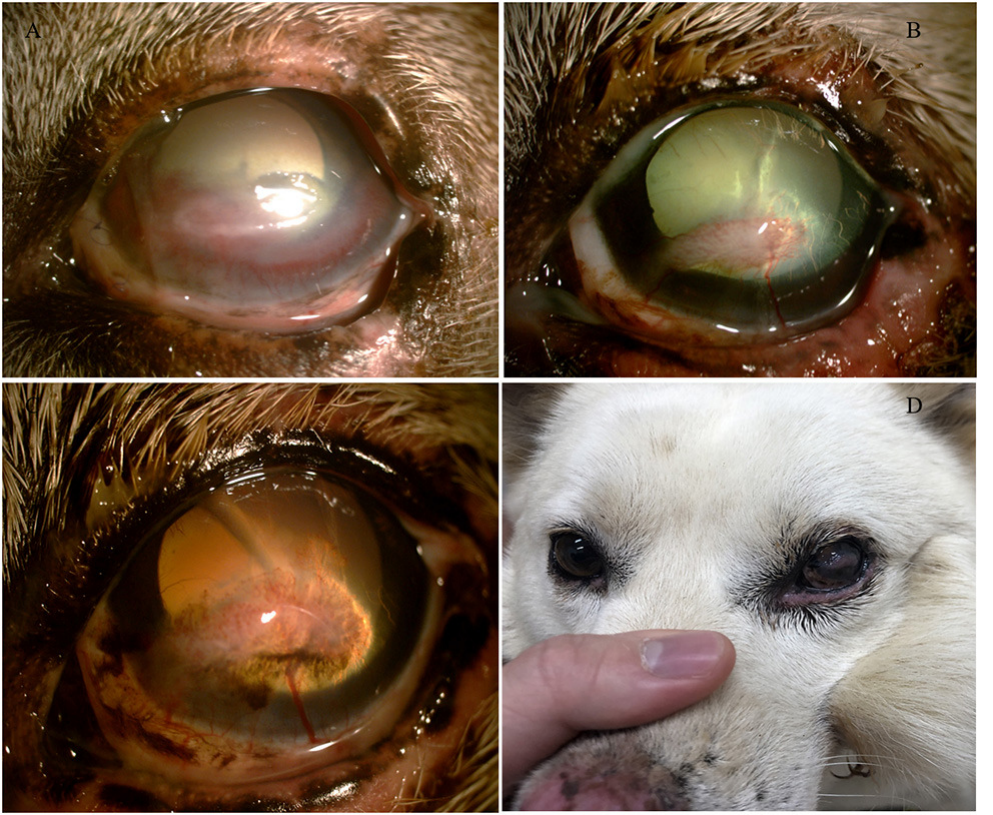
**(A)** The corneal condition 21 days after surgery. **(B)** The corneal condition 7 weeks after surgery. **(C)** The corneal condition 1.5 years after surgery. **(D)** Globe positions of both eyes 1.5 years after surgery.

Although the globe was located in a slightly dorsolateral position, the ocular malposition was improved (Figure 4B). Some sutures at the flap and donor site were removed 21 days post-operatively (Figure 4C). The menace response and the palpebral reflexes were normal OU, but a corneal ulcer with superficial and deep neovascularization was noted (Figure 5A). On ophthalmologic examination OS, Schirmer Tear Test (STT) was 11 mm/min, and the intraocular pressure (IOP) was 13 mmHg. Topical eye drops with antibiotic and artificial tear were applied for 3 weeks. At the follow-up examination 7 weeks post-op, no detectable visual deficits were noted OS. The corneal ulcer was healing with normal STT and IOP (Figure 5B). At the final follow-up examination 1.5 years after surgery, the chronic scar on the cornea and pigmentation at the medial canthus remained (Figure 5C).

This was the first attempt by the authors to repair such a severe proptosis and maxillofacial wound case. Although the local subdermal plexus rotation flap healed well, the repair of proptosis resulted in residual ocular malposition. Additionally, even though the ocular alignment was slightly dorsolateral after the surgery due to the stimulation of the DRM, the globe position was stable with no apparent vision loss. At the follow-up 1.5 years after surgery, the globe position was near normal (Figure 5D).

## Discussion

This case report describes the surgical management of severe facial skin wounds and proptosis due to a traffic accident using a local subdermal plexus rotation flap and transposition of the dorsal rectus muscle with the SIS in a dog. To the best of our knowledge, this partial transposition of the dorsal rectus muscle combined with SIS is the first reported attempt to restore the ocular position due to muscle trauma in dogs.

Due to tension and lack of remaining local skin, primary repair of the defect was not possible. Options to repair the maxillofacial region could include a “lip to lid” flap for replacement of the lower eyelid and/or an axial pattern flap (i.e., STA flap, caudal auricular, etc.) (2, 16). However, the tissue and blood vessels required for these potential flaps were included in the damaged area, preventing these surgical options. As a result, the subdermal plexus rotation flap was used to repair the large skin defect.

In the veterinary literature, length-to-width ratios from 1:1 to 3:1 have been recommended to prevent complications (2). Although flap length can affect the integrity of the flap, sufficient tissue (10 cm width and 22 cm length) was harvested to cover the defect in this case. One common complication of the flap is necrosis to the distal aspect of the flap because of insufficient vascularity, and thus, inadequate blood supply (17). Due to the high density of the subdermal plexus in the face, local skin flaps in the head have been considered safer than other flaps (5). In this case, the local flap healed well at the recipient bed without any complications.

In this case, porcine SIS was used above the torn sclera and MRM. To help with the ocular alignment, the DRM was split and transposed to the SIS so that the dorsolateral force of the eyeball was weakened in this case. Bioscaffolds were needed to provide structural support and a viable blood supply because of the ruptured MRM. The SIS has been studied as a good alternative material with advantages, such as cost-effectiveness, easy handling, and commercial availability (18). Because the SIS was sufficiently durable, the torn regions were well-supported in this case. Porcine SIS, an acellular and biological extracellular matrix (ECM), has been widely used to support ECM deficits (19). In addition, it has been reported to significantly increase the rate of wound healing (19). In veterinary medicine, the SIS has been mostly used as corneal graft materials in place of frozen or fresh corneal tissue (18).

## Conclusion

This report describes the surgical management of severe proptosis and maxillofacial wounds using a local subdermal plexus flap and partial transposition of the DRM combined with SIS. Despite the extent of the dog's injuries, this novel surgical procedure helped provide good functional and cosmetic results.

## Data Availability Statement

The original contributions presented in the study are included in the article, further inquiries can be directed to the corresponding author/s.

## Ethics Statement

Written informed consent was obtained from the owners for the participation of their animals in this study.

## Author Contributions

JuK and MK reviewed and edited the manuscript. DK, JaK, DS, HH, YK, TC, SL, and HL collected data. All authors contributed to data interpretation and preparation of the manuscript.

## Conflict of Interest

The authors declare that the research was conducted in the absence of any commercial or financial relationships that could be construed as a potential conflict of interest.
